# Age-Related CT Morphometry of the Pediatric Clivus and Dorsum Sellae: Cross-Sectional Changes and Pneumatization Patterns

**DOI:** 10.3390/diagnostics16193074

**Published:** 2026-09-22

**Authors:** Koray Bingöl, Zeynep Betül Deve

**Affiliations:** Department of Anatomy, Erzincan Binali Yıldırım University, Erzincan 24002, Turkey; koray.bingol@erzincan.edu.tr

**Keywords:** dorsum sellae, clivus, pediatric, computed tomography, morphometry, pneumatization, skull base, developmental anatomy

## Abstract

**Background/Objectives:** This study investigated age-related morphometric and angular changes in the dorsum sellae and clivus, and evaluated dorsum sellae pneumatization patterns in a pediatric population using computed tomography (CT). **Methods:** This retrospective study included 720 pediatric subjects aged 0–18 years who underwent cranial CT. Subjects were stratified into five age groups. Four parameters were evaluated on multiplanar reformatted midsagittal images: clivus length, dorsum sellae middle thickness (ML-T), sphenoid–dorsal angle (SDA), and sellar–clival inclination angle (SCIA). Dorsum sellae pneumatization was classified into four types (Types 0–3) according to the criteria of Alpergin et al. **Results:** Clivus length increased with age, from 32.11 ± 5.65 mm in participants aged 0–2 years to 46.96 ± 3.67 mm in those aged 14–18 years. The SDA decreased from 103.49 ± 3.54° to 89.95 ± 9.86° and ML-T decreased from 3.95 ± 0.40 mm to 2.12 ± 0.61 mm. The SCIA differed between age groups but showed no consistent linear trend with age (r = −0.005, *p* = 0.884). In unadjusted comparisons, male subjects had a slightly greater clivus length. ML-T, SDA, and SCIA did not differ significantly between sexes. Following adjustment for age and the age × sex interaction, sex showed a weak association with ML-T (standardized β = 0.111, *p* = 0.007) but not with other morphometric parameters. Type 0 pneumatization was universal from 0 to 9 years of age. Pneumatization was observed in 48.6% of children aged 10–13 years and 34.0% in those aged 14–18 years; however, Type 2 and 3 patterns occurred exclusively in the 14–18-year group, indicating higher-grade pneumatization rather than a monotonic increase in overall prevalence. **Conclusions:** In this pediatric cohort, clivus length increased with age, whereas SDA and ML-T decreased. This study provides age group-specific descriptive morphometric data with excellent interobserver reproducibility; however, the findings should not be interpreted as normative reference standards.

## 1. Introduction

The dorsum sellae is a key osseous landmark of the sellar region, forming the posterior boundary of the sella turcica and contributing to posterior clinoid processes. Due to its close relationship with the pituitary stalk and adjacent sellar and parasellar structures, precise knowledge of its morphology is critical for radiologic interpretation and skull base surgery. The clivus, which extends inferiorly from the sellar region as the sloping posterior skull base, is anatomically continuous with the dorsum sellae and also represents an important structure in imaging-based evaluation and surgical planning of the central skull base [1,2].

Developmental changes in the posterior sellar region occur throughout childhood. Berger et al. noted that evaluation of the dorsum sellae in infants and children may be challenging as normal development can produce variations in the shape of the dorsum and posterior clinoid processes which mimic pathology [1]. Similarly, Kumar and Govindraju demonstrated that sella turcica morphology varies with age, underscoring the need for age-specific interpretation of sellar anatomy [3]. More recently, a pediatric computed tomography (CT) study showed that dorsum sellae dimensions change throughout childhood and adolescence, with pneumatization increasingly prevalent with advancing age [4]. As pneumatization of the dorsum sellae and adjacent clivus can alter the contour and internal density of the posterior sellar wall, awareness of the normal developmental timing of this process is clinically important, as it may otherwise be mistaken for an osseous lesion or a destructive process on pediatric imaging.

The dorsum sellae also has considerable clinical relevance beyond its developmental anatomy. Gulsen et al. demonstrated that the location of the pituitary stalk relative to the dorsum sellae and adjacent sellar landmarks is variable, underlining the importance of precise regional anatomy [2]. CT-based studies by Ilków et al. and Alpergin et al. highlighted that sphenoid sinus pneumatization may extend into the dorsum sellae and posterior clinoid processes and proposed practical imaging-based patterns for dorsum sellae pneumatization [5,6,7]. These studies indicate that normal morphologic variation in the posterior sellar wall should be carefully considered during radiological assessment and surgical approaches involving the sellar and parasellar regions [2,3,4,5]. Providing age-specific descriptive data for these structures may therefore assist the accurate differentiation of normal developmental changes from pathological alterations when evaluating the pediatric skull base.

However, comprehensive pediatric data integrating linear morphometry, angular variation, and dorsum sellae pneumatization within the same population across the 0–18-year age range remain limited. The value of the present study is therefore not the introduction of an isolated new morphometric parameter, but the simultaneous evaluation of complementary structural features of the posterior sellar and clival regions in a large, age-stratified pediatric cohort. This approach facilitates the characterization of age-related differences in clival length, dorsum sellae thickness, angular measurements, and pneumatization severity within a single cross-sectional framework.

## 2. Materials and Methods

### 2.1. Study Population

This retrospective cross-sectional study included pediatric patients aged 0–18 years who underwent clinically indicated non-contrast cranial CT between January 2024 and December 2025. All eligible examinations were retrospectively screened from the institutional Picture Archiving and Communication System. Ethical approval was obtained from the Non-Interventional Clinical Research Ethics Committee (meeting no.: 09; decision no.: 2026-09/03; approval date: 7 May 2026).

Patients were eligible for inclusion if aged 0–18 years at the time of CT acquisition; there were no craniofacial, sellar, spheno-occipital, or central skull base abnormalities which could affect the evaluated measurements; the image quality permitted clear visualization of the dorsum sellae, sphenoid sinus, clivus, and anterior margin of the foramen magnum; and there was availability of thin-section images suitable for multiplanar reconstruction.

The exclusion criteria included previous cranial/skull base surgery; craniofacial trauma involving the central skull base; congenital craniofacial/skull base anomalies; skeletal dysplasia or systemic diseases known to affect bone development; intracranial mass, hydrocephalus, or any other abnormality causing distortion of the sellar or clival region; significant motion, beam-hardening, or reconstruction artifacts; and repeat examinations for the same patient.

A total of 3165 cranial CT scans performed during the study period were screened. Of these, 2008 were excluded due to previous cranial or skull base surgery (*n* = 97); central skull base trauma (*n* = 431); congenital craniofacial/skull base anomalies (*n* = 126); skeletal dysplasia or systemic diseases affecting bone development (*n* = 63); intracranial mass, hydrocephalus, or other abnormalities distorting the sellar or clival region (*n* = 521); significant imaging artifacts (*n* = 238); and repeat examinations for the same patient (*n* = 532). After application of the eligibility criteria, 1157 subjects remained eligible.

As the number of eligible subjects differed between the predefined developmental age strata, age-stratified random sampling was performed to obtain equal-sized groups. Within each age category, subjects were assigned computer-generated random numbers and 144 subjects were randomly selected. This yielded a final cohort of 720 subjects. The remaining 437 otherwise eligible subjects were excluded solely due to the sampling procedure. For descriptive comparisons, participants were divided into five age categories: 0–2 years; 3–5 years; 6–9 years; 10–13 years; 14–18 years (*n* = 144, each).

The cohort comprised 396 males and 324 females. Age categories were used for descriptive presentation and group comparisons. Exact age at CT acquisition was retained as a continuous variable for regression analyses. To ensure balanced developmental comparisons, equal numbers of subjects were included in each age category (Figure 1). The indications for CT scans for the included subjects were recorded.

### 2.2. CT Protocol and Image Analysis

All CT scans were acquired using the same dual-source 128-slice CT system (SOMATOM Definition Flash, Siemens Healthineers, Forchheim, Germany) and the institutional non-contrast pediatric cranial CT acquisition framework. The basic acquisition and reconstruction settings were consistent across examinations; however, radiation exposure was not fixed at a single mAs value. Effective mAs was adjusted between 150 and 225 according to patient age and size per the ALARA principle. Tube voltage was 140 kVp. The field of view ranged from 150 to 300 mm according to patient size, with a 512 × 512 acquisition matrix and a reconstructed slice thickness of 1.0 mm.

These parameters were selected to provide adequate visualization of the small osseous structures of the central skull base. The combination of a 512 × 512 matrix and a 150–300 mm field of view yielded an approximate in-plane pixel size of 0.29–0.59 mm, while the 1.0 mm thin-section reconstructions limited partial volume averaging during multiplanar morphometric measurements. The relatively high tube potential provided adequate photon penetration through the dense skull base and helped reduce beam-hardening artifacts. Age- and size-adjusted tube current exposure was used to balance image noise and radiation dose. Only scans in which the dorsum sellae, clivus, sphenoid sinus, and anterior margin of the foramen magnum were clearly delineated were included in the analysis.

Images were reconstructed in axial, coronal, and sagittal planes. Multiplanar reformatted (MPR) images were generated and midsagittal and coronal images were used for all measurements. All images were transferred to an advanced workstation (Syngo.via, version VB60S_HF04, Siemens Healthineers, Erlangen, Germany) for detailed evaluation.

### 2.3. Measured Parameters

All morphometric and angular measurements were performed on high-resolution midsagittal MPR images using standardized anatomical landmarks. Reference lines and measurement points were consistently placed along well-defined osseous boundaries to minimize variability related to landmark selection. For each examination, the sagittal image most clearly showing the sellar floor, dorsum sellae, clivus, and anterior margin of the foramen magnum was used for measurement. Linear and angular measurements were obtained using the electronic tools of the workstation. Representative examples of the measurements are illustrated in Figure 2 and Figure 3.

Four parameters relating to the morphology of the dorsum sellae and its spatial relationship with adjacent skull base structures were evaluated on midsagittal CT images:

- **Middle thickness** (**ML-T**): the anteroposterior diameter of the dorsum sellae at its midpoint;
- **Clivus length**: the linear distance from the dorsum sellae to the anterior margin of the foramen magnum along the clival surface;
- **Sphenoid–dorsal angle **(**SDA**): the angle between the superior border of the sphenoid sinus and the posterior surface of the dorsum sellae;
- **Sellar–clival inclination angle **(**SCIA**): the angle between the sellar floor and the superior surface of the clivus, representing the inclination of the central skull base.

Potential sources of measurement error included selection of the exact midsagittal plane, placement of linear/angular reference points, partial volume effects at thin osseous margins, and image noise or beam-hardening at the skull base. These effects were minimized through the use of standardized anatomical landmarks, thin-section multiplanar reconstructions, and exclusion of examinations with significant motion, beam-hardening, or reconstruction artifacts. The influence of observer-dependent measurement variability was evaluated by interobserver reliability analysis in a randomly selected subset of the cohort.

### 2.4. Assessment of Pneumatization

Several classification systems have been used to characterize sphenoid sinus and posterior sellar pneumatization. Earlier systems, including the Hardy classification, primarily categorize the overall extension of sphenoid sinus pneumatization in relation to the sella turcica and may distinguish postsellar extension involving the dorsum sellae and posterior clinoid processes [6]. The present study used the system proposed by Alpergin et al. [5] which specifically grades the extent of pneumatization within the dorsum sellae itself rather than the general configuration of the sphenoid sinus.

Thus, pneumatization was classified as follows: Type 0, no pneumatization; Type 1, pneumatization <50% of the height of the dorsum sellae; Type 2, pneumatization >50% but incomplete; and Type 3, complete pneumatization reaching the upper border of the dorsum sellae. This represents a simple ordinal assessment of dorsum sellae involvement, distinguishing the absence of pneumatization from limited (<50%), advanced (>50%), and complete involvement. This was considered particularly suitable for this developmental analysis as it facilitates direct comparison of pneumatization severity across pediatric age groups.

Pneumatization was evaluated on midsagittal CT images based on its extent relative to the inferior border, longitudinal axis, and superior tip of the dorsum sellae. All assessments were performed by the authors. Representative examples are illustrated in Figure 4.

### 2.5. Statistical Analysis

Statistical analyses were performed using IBM SPSS Statistics version 29.0 (IBM Corp., Armonk, NY, USA) and R software version 4.4.0 (R Foundation for Statistical Computing, Vienna, Austria). A two-sided *p* value < 0.05 was considered significant unless otherwise specified.

The distribution of continuous variables was assessed using histograms, Q–Q plots, Shapiro–Wilk tests, and skewness and kurtosis statistics. Due to its sensitivity to minor deviations from normality in relatively large samples, normality was not determined solely by the Shapiro–Wilk test; graphical appearance, skewness and kurtosis, and formal normality testing were jointly considered. The morphometric variables showed distributions sufficiently close to normal for parametric analyses, without marked skewness or extreme departures on Q–Q plots. Accordingly, continuous variables are presented as mean ± standard deviation (SD), with parametric procedures used for group comparisons. Categorical variables are presented as frequencies and percentages.

Participants were categorized into five developmental age groups for descriptive analysis. As each age group comprised an equal, relatively large number of subjects (*n* = 144) and the continuous morphometric variables demonstrated approximately normal distributions, between-group differences were compared using parametric analysis of variance (ANOVA). Homogeneity of variances was assessed using Levene’s test. Where homogeneity assumptions were satisfied, conventional one-way ANOVA followed by Tukey’s HSD test for post hoc pairwise comparisons was applied. Where homogeneity assumptions were violated, Welch’s ANOVA followed by Games–Howell multiple comparison testing was applied. For every ANOVA model, the following were reported: F statistic; *p* value; and omega squared (ω^2^) effect size.

### 2.6. Reliability Analysis

To evaluate interobserver measurement reproducibility, approximately 25% of the subjects (*n* = 180) were randomly selected for reliability assessment. All morphometric measurements in this subset were independently performed by two radiologists blinded to each other’s measurements and to patient demographic information.

Interobserver agreement for continuous variables (clivus length, ML-T, SDA, SCIA) was evaluated using a two-way random-effects intraclass correlation coefficient with absolute agreement for single measurements [ICC (2,1)] and 95% confidence intervals (CIs). ICCs were interpreted according to Koo and Li as follows: <0.50, poor; 0.50–0.74, moderate; 0.75–0.89, good; ≥0.90, excellent reliability.

This analysis was conducted to quantify the magnitude of observer-related measurement variability and determine whether landmark placement constituted a significant source of measurement error.

### 2.7. Age-Related Analyses

Age group comparisons were the primary descriptive analyses. Exact age was evaluated as a continuous variable in secondary association analyses. Pearson’s correlation coefficients were calculated for exact age and each morphometric parameter. Secondary multivariable linear models were fitted separately for clivus length, ML-T, SDA, and SCIA using the prespecified structure Y = β0 + β1(age) + β2(sex) + β3(age × sex) + ε.

Unstandardized coefficients (B), standardized beta coefficients (β), standard errors, 95% CIs, *p* values, and adjusted R^2^ values were reported. These models were intended to quantify within-sample linear associations, not generate individual predictions or normative reference limits. No quadratic, restricted cubic spline, or other nonlinear predictive model was retained and no prediction intervals were calculated.

### 2.8. Sex-Related Analyses

Unadjusted differences between male and female subjects were evaluated using Welch’s independent-samples *t*-test and are reported as crude comparisons. For the unadjusted comparisons, effect size was expressed as Cohen’s d with 95% CIs.

As the male and female groups differed in age distribution and the principal morphometric outcomes were strongly age-dependent, additional multivariable linear regression analyses including age, sex, and age × sex interaction terms were performed. The unadjusted *t*-test and regression sex coefficient were interpreted separately: the former estimates the crude difference in group means; the latter represents the sex association after accounting for age and the interaction term. Adjusted sex associations were interpreted with emphasis on standardized effect size and statistical significance.

### 2.9. Pneumatization Analysis

The association between developmental age group and dorsum sellae pneumatization type was summarized using Pearson’s chi-square statistic. As 10 of the 20 cells (50%) had expected counts below 5, statistical significance was determined using the Fisher–Freeman–Halton exact test with Monte Carlo simulation based on 100,000 samples. The magnitude of the association was quantified using Cramer’s V, calculated from Pearson’s chi-square statistic.

Advanced pneumatization (Type 2 or 3) was observed in only 24 subjects. Therefore, no multivariable binary or ordinal regression model was fitted. The number of advanced cases was insufficient relative to the six candidate predictors, and age was strongly associated with several morphometric variables, particularly clivus length, ML-T, and SDA. Including these variables in the same model would have produced unstable, potentially overfitted estimates. Pneumatization was consequently analyzed using descriptive frequencies and the age group-by-pneumatization type association test described; no independent predictor analysis was performed.

### 2.10. Multiple Testing

As four primary morphometric outcomes were evaluated, multiplicity was controlled using the Holm–Bonferroni procedure. Secondary exploratory analyses were interpreted primarily according to effect sizes and CIs rather than statistical significance alone.

## 3. Results

### 3.1. Study Population Characteristics

A total of 720 pediatric subjects who fulfilled the eligibility criteria were included in the analysis. The cohort comprised 396 males (55.0%) and 324 females (45.0%) with a mean age of 7.87 ± 5.41 years (range: 0–18 years). To facilitate developmental comparisons, subjects were categorized into five age groups (*n* = 144, each). The demographic characteristics of the cohort are summarized in Table 1.

The most common indication for cranial CT was head trauma (335/720; 46.5%), followed by headache (133/720; 18.5%), suspected seizure (106/720; 14.7%), altered level of consciousness (87/720; 12.1%), and vomiting (59/720; 8.2%) (Table 2).

### 3.2. Measurement Reliability

Interobserver agreement in the 180-subject reliability subset was excellent for all morphometric measurements, with ICCs ranging from 0.984 to 0.999. The highest agreement was observed for clivus length; ML-T, SDA, and SCIA also demonstrated excellent reproducibility (Table 3). This indicates that observer-dependent variability was minimal and unlikely to have significantly affected the principal morphometric comparisons.

### 3.3. Age-Related Morphometric Variation

The omnibus age group comparison was significant for clivus length, ML-T, SDA, and SCIA (all *p* < 0.001) (Table 4); however, the age-related pattern differed between the parameters.

Clivus length increased progressively across consecutive age groups, whereas ML-T showed a gradual reduction with advancing age. SDA also demonstrated a continuous decrease throughout childhood and adolescence. For SCIA, the omnibus ANOVA was significant but the group means fluctuated nonmonotonically (58.18°, 54.26°, 58.03°, 55.15°, and 57.56° across the successive age groups) rather than showing a progressive increase or decrease. Accordingly, the SCIA result was interpreted as between-group heterogeneity without a consistent cross-sectional age-related trend. Its effect size (ω^2^ = 0.145) was lower than those of the clivus length, ML-T, and SDA.

Of the evaluated variables, clivus length exhibited the largest age-related effect (ω^2^ = 0.699), followed by ML-T (ω^2^ = 0.642) and SDA (ω^2^ = 0.501) (Table 4).

### 3.4. Sex-Related Differences

Comparisons between male and female subjects are presented in Table 5. In the unadjusted comparisons, male subjects had a slightly greater clivus length than females although the corresponding effect size was small. No crude between-sex differences were observed for ML-T, SDA, or SCIA. These unadjusted findings were considered separately from the age-adjusted regression results as the male group was older on average and ML-T, in particular, was strongly associated with age.

### 3.5. Correlation Between Age and Morphometric Parameters

Correlation analysis demonstrated a strong positive association between age and clivus length (r = 0.808; *p* < 0.001) and strong negative associations between age and ML-T (r = −0.773; *p* < 0.001) and SDA (r = −0.663; *p* < 0.001). SCIA showed no meaningful linear correlation with age (r = −0.005; *p* = 0.884) (Table 6). This near-zero correlation is not inconsistent with the significant omnibus ANOVA: ANOVA tests whether any age group means differ, whereas Pearson’s correlation evaluates a linear age-related trend.

### 3.6. Secondary Multivariable Linear Association Analyses

The secondary multivariable linear association analyses are summarized in Table 7. Age was strongly associated with clivus length, ML-T, and SDA after adjustment for sex and the age × sex interaction term (all *p* < 0.001). The models explained a substantial proportion of within-sample variance for clivus length (adjusted R^2^ = 0.653) and ML-T (adjusted R^2^ = 0.605) and a moderate proportion for SDA (adjusted R^2^ = 0.437). No linear association between age and SCIA was identified. These estimates were interpreted as association measures and were not used to derive individual predicted values or reference intervals.

### 3.7. Distribution of Dorsum Sellae Pneumatization

Pneumatization type was significantly associated with age group (Pearson’s χ^2^ = 328.968; Fisher–Freeman–Halton Monte Carlo exact *p* < 0.001; Cramer’s V = 0.390) (Table 8). Type 0 pneumatization was the only pattern observed from 0 to 9 years of age. Any pneumatization was present in 70 of 144 children (48.6%) aged 10–13 years and 49 of 144 children (34.0%) aged 14–18 years; therefore, the overall prevalence did not increase monotonically across the two oldest age groups. However, the distribution shifted toward greater severity with age: all pneumatized cases in the 10–13-year group were Type 1, with Type 2 and 3 patterns identified exclusively in the 14–18-year group.

### 3.8. Advanced Pneumatization Patterns

Type 2 or 3 pneumatization was identified in only 24 subjects, all of whom were aged 14–18 years. Due to the limited number of advanced cases and the strong associations between age and several morphometric parameters, a multivariable regression model was not considered statistically reliable and was not retained. Accordingly, the findings are reported descriptively and no variable is interpreted as an independent predictor of advanced pneumatization.

## 4. Discussion

The key contribution of this study is the integrated cross-sectional characterization of age-related variation in the posterior sellar and clival regions across the pediatric age range. Rather than investigating a single dimension or pneumatization pattern in isolation, clivus length, dorsum sellae thickness, two angular relationships, and dorsum sellae pneumatization, variables not captured together in previous pediatric studies, were simultaneously evaluated in 720 children across five age groups. Older age groups had greater clival length and lower ML-T and SDA values; SCIA demonstrated nonmonotonic variation and advanced dorsum sellae pneumatization was restricted to adolescence. Thus, these findings extend previous morphometric observations by demonstrating that linear dimensions, angular configuration, and pneumatization show distinct cross-sectional age-related patterns rather than uniform age-associated variations.

It should be emphasized that the principal quantitative findings of this study, including age-related patterns of clival length, ML-T, SDA, and pneumatization, are derived directly from measurements obtained in the present pediatric cohort. Where studies on adult populations are cited for comparison or surgical context, these serve only to situate these pediatric findings within the broader literature of the field and should not be interpreted as pediatric evidence in themselves.

The study by Atadağ et al. [4] represents the closest pediatric CT comparison to the present work. In the cohort of 360 children aged 1–18 years, several dimensions of the dorsum sellae (including ML-T, an angular parameter, and the same four-level pneumatization classification) were evaluated. Thus, the present study may be regarded as an incremental anatomical and methodological extension of that work and not the first broad age-related characterization of the pediatric dorsum sellae. This cohort was twice as large (*n* = 720), included subjects from infancy through 18 years of age, and utilized equal-sized age strata. More importantly, the analysis extended beyond the dorsum sellae itself by integrating clivus length with ML-T, SDA, SCIA, and dorsum sellae pneumatization, allowing the posterior sellar and clival regions to be assessed as anatomically related components of the central skull base. Exact age was retained for secondary continuous age association analyses, complementing the categorical age group comparisons.

An important difference between the two studies concerns ML-T: Atadağ et al. reported an initial decrease followed by an increase at older ages, whereas ML-T in this cohort was progressively lower across older age groups and showed a strong negative cross-sectional association with age. This discrepancy may reflect differences in age stratification, cohort composition, sampling strategy, and technical- or landmark-related aspects of CT measurement. In particular, grouping subjects into relatively broad developmental age strata, as in the present study, may smooth narrower age-specific fluctuations detectible with alternative age-grouping schemes. Thus, the differing ML-T patterns should not be interpreted as mutually exclusive developmental models, but rather as evidence that dorsum sellae thickness may show population- and methodology-dependent variability warranting confirmation in multicenter cohorts using harmonized measurement protocols.

The most notable finding was the progressive increase in the clival length during childhood. Similar observations and related anatomical findings are reported in other anatomic/cephalometric studies of the clivus and adjacent skull base structures [8,9,10,11]. Hedayatian et al. reported a significant positive association between clival length and age, with Serindere et al. highlighting its morphometric variability and surgical importance in adults [11,12]. The findings of this study are consistent with these observations and show a robust cross-sectional association between age and clival length; however, the linear model retained was not developed or validated as a predictive formula for individuals.

Elongation of the clivus is a sign of coordinated growth of the central cranial base, not of isolated expansion of a single structure. The spheno-occipital synchondrosis is a major growth center during childhood and alters spatial relationships between the sphenoid bone, dorsum sellae, occipital bone, and craniovertebral junction [10,13]. These changes may be responsible for the concomitant angular change and progressive sphenoid sinus pneumatization associated with clival elongation in this cohort.

Clival length had the largest effect size of all evaluated variables, indicating that clival growth represents one of the dominant morphologic events of pediatric skull base maturation. The reported group means and SDs may assist age-aware interpretation but should not be considered as fixed normative thresholds. Accurate knowledge of normal clival development remains relevant to preoperative planning of transclival and endoscopic endonasal approaches where small anatomical differences can affect surgical corridors and neurovascular relationships [9,12,13]. The secondary regression analyses quantified continuous age associations but did not establish validated reference equations or individual prediction limits.

### 4.1. Age-Related Morphometric and Angular Variation in the Posterior Sellar Region

In addition to the cross-sectional age association of clival length, this study revealed age-related differences in the geometric configuration of the posterior sellar region. SDA and ML-T were lower in older age groups, indicating that the posterior sellar architecture differs considerably across pediatric age categories. Postnatal ossification of the dorsum sellae was described by Berger et al. [1], with systematic age-related morphological differences on pediatric CT reported by Atadağ et al. [4]. Considered with previous studies on postnatal clival development [10] and skull base growth [13], these cross-sectional observations are consistent with significant age-related variation in posterior sellar anatomy throughout childhood and adolescence and support the concept that the posterior sellar region remains developmentally active across this period.

The progressive decrease in SDA may reflect posterior reorientation of the dorsum sellae accompanying clival elongation, sphenoid body remodeling, and increasing sphenoid sinus pneumatization. Postnatal clival growth relates primarily to endochondral ossification at the spheno-occipital synchondrosis, accompanied by changing spatial relationships between the sphenoid, basiocciput, sellar floor, and craniovertebral junction [10,13]. Previous CT studies have demonstrated substantial interindividual variability in dorsum sellae and posterior clinoid pneumatization [5,6,14]. Pediatric imaging studies have further shown that sphenoid sinus pneumatization develops progressively during childhood and is generally well established by late childhood or early adolescence [15,16]. The decline in SDA with age may be an aspect of the remodeling needed to accommodate both growth of the skull base and progressive aeration of the sphenoid.

The decrease in ML-T further suggests that dorsum sellae maturation is not characterized solely by expansion. Its apparent thinning with age may result from cortical remodeling combined with progressive posterior extension of sphenoid sinus pneumatization, consistent with previous CT studies of age-related changes in posterior clinoid morphology [7]. However, as volumetric or cortical thickness analyses were not performed, this could not be directly assessed.

In contrast, SCIA demonstrated significant between-group heterogeneity without a consistent age-dependent trend. The means alternated across the age groups, with both Pearson’s correlation and linear regression showing no clear linear association with age. These results are methodologically compatible as the omnibus ANOVA detects differences between group means (including nonmonotonic differences), whereas correlation and linear regression evaluate directional linear change. SCIA should therefore not be interpreted as progressively increasing or decreasing during childhood. Rather, the observed pattern may reflect individual or developmental stage-specific architectural variation in the sellar–clival relationship, consistent with Chen et al.’s observation that different components of the pediatric skull base do not mature uniformly [13].

The sex-related findings also require a distinction between crude and adjusted analyses. In the unadjusted comparison, male and female subjects had identical mean ML-T values (3.03 mm; *p* = 0.979) but the male group was older on average and ML-T decreased strongly with age. After adjustment for age and the age × sex interaction, sex showed a significant but small association with ML-T (standardized β = 0.111; *p* = 0.007) and the interaction term remained nonsignificant. This should therefore be interpreted as a modest age-adjusted association and not evidence of a large or clinically established sexual dimorphism. No adjusted sex association was detected for clivus length, SDA, or SCIA.

The absence of a consistent linear age trend for SCIA may also have biomechanical significance: growth at the spheno-occipital synchondrosis produces longitudinal expansion but surrounding structures remodel coordinately to preserve alignment with the brainstem, pituitary region, and craniovertebral junction [10,13]. SCIA may therefore function less as a linear marker of chronological growth than as an individual or developmental stage-specific architectural characteristic, consistent with radioanatomic studies showing that the feasibility of pediatric endoscopic endonasal approaches depends on specific anatomical corridors—including sphenoid pneumatization, clival dimensions, working distances, and intercarotid relationships—rather than age alone [13,17,18].

Given the nonmonotonic variation in SCIA across age groups, the present dataset does not permit a reliable assessment of whether SCIA is independently associated with advanced dorsum sellae pneumatization. Only 24 subjects demonstrated Type 2 or 3 patterns, and age was strongly correlated with several morphometric variables. A multivariable model including these interrelated predictors would therefore be vulnerable to overfitting and unstable coefficients. Any potential relationship between sellar–clival geometry and pneumatization should be regarded as unproven and validated in larger cohorts with adequate numbers of advanced cases.

The continuous age analyses were retained as secondary association analyses complementing the age group comparisons by quantifying the direction and magnitude of linear relationships. They were not designed as normative prediction models. As fitted predictive equations, validated nonlinear trajectories, percentile curves, and prediction intervals were not developed, these findings should be interpreted as descriptive cross-sectional age-related data rather than patient-level reference standards. Formal normative modeling will require larger multicenter cohorts, external validation, and age- and sex-specific percentile or prediction limits.

From a clinical perspective, these findings may assist radiologists in distinguishing expected developmental anatomy from potentially abnormal osseous morphology. The marked increase in clivus length and the age-related decreases in ML-T and SDA indicate that a measurement which appears unusual when considered without age context may represent normal developmental variation [1,4,10,13]. Awareness of these patterns is particularly critical when evaluating the pediatric sellar and central skull base region for suspected congenital abnormality, osseous remodeling, or destructive disease [1,4]. However, as these data are descriptive and were not developed as validated reference intervals, the reported age group values should be treated as contextual information, not as diagnostic cutoff values.

These findings also hold potential relevance for preoperative CT assessment of the central skull base. Clival dimensions, dorsum sellae thickness, sellar–clival geometry, and the extent of sphenoid/dorsum sellae pneumatization may influence the amount and orientation of bone encountered during posterior clinoidectomy and transsellar or transclival approaches [9,12,13,17,18].

In particular, recognition of advanced dorsum sellae pneumatization is vital as opening a pneumatized posterior sellar structure during drilling may establish communication with the sphenoid sinus and increase the risk of cerebrospinal fluid leakage, infection, or an inadequately sealed air cell tract and may also reduce the compact bone available for controlled drilling. Thin-section multiplanar CT should therefore document the presence, extent, and route of pneumatization before surgery involving these structures [5,7,14]. Age-aware evaluation of these anatomical features may also improve anticipation of developmental constraints and facilitate individualized surgical planning [13,17,18,19]; however, the present measurements alone cannot determine procedural feasibility.

Overall, these cross-sectional data demonstrate distinct age-related patterns in posterior sellar and clival morphology. Older age was associated with greater clival length and lower SDA and ML-T values, whereas SCIA showed nonmonotonic age group variation without a linear age relationship. The confinement of Type 2 and 3 pneumatization to the oldest age group indicates age-related differences in pneumatization severity. These findings characterize cross-sectional developmental patterns and should not be interpreted as directly observed longitudinal changes within individual children.

### 4.2. Dorsum Sellae Pneumatization and Its Developmental Significance

Another major finding was an age-related shift in the severity of dorsum sellae pneumatization rather than a monotonic increase in its overall prevalence. Any pneumatization was more frequent in the 10–13-year group than the 14–18-year group; however, all pneumatized cases at 10–13 years were limited to Type 1; the more advanced Type 2 and 3 patterns occurred exclusively in adolescents aged 14–18 years. This distribution supports the interpretation that posterior extension into the dorsum sellae represents a relatively late component of sphenoid sinus development. Previous pediatric imaging studies have shown that sphenoid sinus pneumatization begins after early marrow conversion, progresses considerably during childhood, and is generally well developed by 10–14 years [15,16,20]. The present findings are consistent with this developmental model and indicate, on cross-sectional analysis, that older age is more strongly associated with greater pneumatization severity than a steady increase in overall prevalence.

The CT acquisition protocol used in this study was broadly comparable with previous CT-based morphometric and pneumatization investigations in its use of thin-section imaging and multiplanar evaluation; however, technical differences exist. Ilków et al. [6], for example, evaluated dorsum sellae and posterior clinoid pneumatization using 0.625 mm sections acquired at 120 kV on a 16-row CT system, whereas the present study used 1.0 mm reconstructions at 140 kVp on a 128-slice dual-source system. Despite these differences, both protocols provide high spatial resolution datasets suitable for sagittal and coronal assessment of small skull base osseous structures. Other CT-based studies of sphenoid and sellar morphology have similarly used thin-section acquisitions in the approximate 0.6–1.0 mm range, supporting thin-section multiplanar CT as an appropriate technique for such morphometric evaluations. Nevertheless, differences in scanner generation, tube potential, tube current settings, and reconstruction thickness should be considered when comparing quantitative measures across studies.

The biological sequence is thought to begin with the conversion of red to fatty marrow within the presphenoid, followed by resorption and invasion of respiratory epithelium from the developing sphenoid sinus. MR imaging studies have shown that marrow conversion precedes visible pneumatization and may begin within the first year of life, whereas true sinus aeration becomes increasingly apparent during the preschool and school-age periods [20,21]. As the sinus enlarges posteriorly, pneumatization may progressively extend toward the sellar floor, clivus, dorsum sellae, and posterior clinoid processes. This may be a plausible explanation for the parallel reduction in ML-T and increasing pneumatization severity observed in this cohort.

These findings align with previous CT-based studies reporting substantial variability in posterior sellar aeration. Alpergin et al. classified dorsum sellae pneumatization by the proportion of osseous involvement and emphasized its surgical relevance [5]; Ilków et al. noted considerable inter-individual variability in pneumatization extent [6]; and Burulday et al. reported comparable variability in posterior clinoid pneumatization [14]. Notably, a recent pediatric study by Alpergin et al. found posterior clinoid pneumatization in 8.9% of examined sides, significantly associated with age but not sex or laterality [7], with most pneumatized processes detected after late childhood—paralleling the distribution observed here.

Different approaches have been proposed for characterizing pneumatization of the sphenoid and posterior sellar region. The Hardy classification and its applications characterize sphenoid sinus pneumatization according to its relationship with the sella, including postsellar extension toward the dorsum sellae and posterior clinoid processes [6]. Such classifications are particularly useful for describing the overall anatomical configuration and potential surgical corridor of the sphenoid sinus. In contrast, the system proposed by Alpergin et al. [5] focuses on the proportion of the dorsum sellae involved by pneumatization. For the present study, this direct extent-based approach was advantageous as the primary objective was to investigate age-related morphologic variation in the dorsum sellae itself. The ordinal Type 0–3 system also allowed for detection of increasing pneumatization severity even where the overall prevalence did not increase monotonically with age.

Sex-specific differences in dorsum sellae pneumatization could not be assessed reliably in this dataset as the advanced categories comprised only 24 subjects and the sex distribution was not perfectly balanced across age groups. As no stable multivariable model could be fitted, no conclusion regarding an independent effect of sex on pneumatization should be drawn from these data. Larger age- and sex-balanced cohorts are required to evaluate this question.

Advanced Type 2 and 3 patterns were observed only in the 14–18-year age group, supporting a clear descriptive association between age and pneumatization severity. However, as the number of advanced cases was small and multivariable modeling was not statistically reliable, age should not be described as an independent predictor in this cohort. Earlier imaging studies have also demonstrated marked inter-individual variability in the timing and extent of sphenoid sinus development in children of similar ages [15,16,20]. Age-specific distributions may therefore be more clinically informative than rigid age thresholds.

This analysis does not establish an independent relationship between SCIA and advanced pneumatization. Although such a geometric association may be biologically plausible, the small number of advanced cases and collinearity among age-related morphometric variables preclude reliable estimation of its magnitude or direction. This should be investigated in substantially larger, preferably multicenter cohorts using prespecified parsimonious models or penalized regression methods.

Pneumatization can simultaneously facilitate and complicate endoscopic access. A well-pneumatized sphenoid sinus may provide clearer landmarks and reduce the bone that must be removed to reach the sellar or clival target, whereas irregular or extensive recesses may closely approximate critical neurovascular structures and require meticulous closure. Vaezi et al. demonstrated that sphenoid pneumatization predicts surgical corridor dimensions and relationships of the vidian canal and foramen rotundum [22], and lateral/posterior pneumatization patterns are shown to influence the visibility and vulnerability of adjacent skull base structures [23]. Thus, the surgical relevance of aeration depends on the intended corridor rather than being simply favorable or unfavorable.

These considerations are particularly important in pediatric endoscopic endonasal surgery, where the surgical corridor changes continuously with growth. Younger children have less sphenoid aeration, narrower piriform apertures, and less working space, whereas adolescents approach adult skull base proportions [13,18,19]. A CT study of 506 pediatric patients demonstrated significant age-related variation in sphenoid pneumatization relevant to endonasal access [13]. Banu et al. showed that the volume of the sphenoid sinus increases with age and correlates with pneumatization type [18]. However, patient-specific measures such as working distance and intercarotid distance may be more predictive of operative difficulty than age alone and should be considered when determining the feasibility of surgery [24].

These results support systematic preoperative assessment of both external skull base geometry and internal pneumatization, including clival length and thickness, sellar–clival angular relationships, extent of sphenoid and dorsum sellae aeration, posterior clinoid involvement, septal attachment, and the relationship of pneumatized recesses to the internal carotid arteries. Although the parameters evaluated here cannot replace comprehensive preoperative mapping, they may provide objective supplementary markers for identifying patients with more advanced posterior sellar aeration [19].

Overall, dorsum sellae pneumatization appears to be a relatively late component of skull base maturation, with the advanced patterns restricted to adolescence in this cohort. The combination of age-specific pneumatization distributions, angular measurements, and clival morphometry provides a descriptive representation of pediatric posterior sellar anatomy; however, independent contributions of age, sex, or sellar–clival geometry cannot be determined from the limited number of advanced cases.

### 4.3. Strengths, Limitations, and Future Directions

This study has several strengths: a large, well-balanced pediatric cohort (720 subjects ranging in age from infancy to adolescence); simultaneous assessment of linear, angular, and pneumatization-related parameters within the same dataset; excellent interobserver reliability (ICC 0.984–0.999) supporting reproducible measurement on the midsagittal MPR CT; and a statistical approach comprising age group comparisons, effect size estimation, correlation, and secondary linear association analyses.

Nevertheless, certain limitations remain. The retrospective, cross-sectional, single-center design precluded direct observation of individual developmental trajectories, with generalizability limited to children receiving clinically indicated CT. Chronological age, rather than pubertal stage or bone age, was used to index maturation and measurements were confined to midsagittal two-dimensional planes. Moreover, although the excellent interobserver ICCs indicate high reproducibility, small systematic errors relating to midsagittal plane selection, cortical boundary definition, and finite CT spatial resolution cannot be entirely excluded. Thus, the reliability results should be interpreted as evidence of measurement reproducibility and not absolute accuracy. Reliability assessment was limited to interobserver ICCs for continuous measurements; intraobserver repeatability, weighted kappa for the pneumatization categories, and Bland–Altman bias analyses were not included in the final reported analysis. Finally, this study did not generate externally validated full prediction equations, nonlinear developmental trajectories, percentile curves, or prediction intervals. Accordingly, the reported age group means and SDs should be interpreted as descriptive morphometric data rather than normative reference limits.

Future research should prioritize prospective, multicenter studies establishing sex- and age-specific percentile curves and fully specified prediction models, ideally incorporating longitudinal imaging, bone age or pubertal stage assessment, nonlinear modeling where supported, and three-dimensional volumetric segmentation of the sphenoid sinus, dorsum sellae, clivus, and posterior clinoid processes. Such studies should report complete model equations, diagnostic performance, prediction intervals, and external validations before the measurements are implemented as clinical reference standards.

## 5. Conclusions

In this cross-sectional pediatric cohort, clival length increased with age, whereas SDA and ML-T decreased. Clival length showed the largest age-related effect of the evaluated morphometric parameters. SCIA differed between age groups but showed neither a monotonic pattern nor a meaningful linear relationship with age and should therefore not be interpreted as a progressive developmental marker.

Dorsum sellae pneumatization exhibited an age-related shift in severity, with Type 2 and 3 patterns occurring only during adolescence. As advanced pneumatization was present in only 24 subjects and several candidate predictors were strongly correlated with age, no reliable independent predictor analysis could be performed. The findings should therefore be interpreted as descriptive cross-sectional age-related patterns rather than evidence that age, SCIA, or another morphometric variable independently determines advanced pneumatization.

The presented CT-based measurements provide descriptive, age group-specific information which may support radiological interpretation and patient-specific preoperative assessment in pediatric skull base imaging. They should not be regarded as normative reference limits until validated percentile curves, complete predictive models, and prediction intervals are established in independent multicenter cohorts.

## Figures and Tables

**Figure 1:**
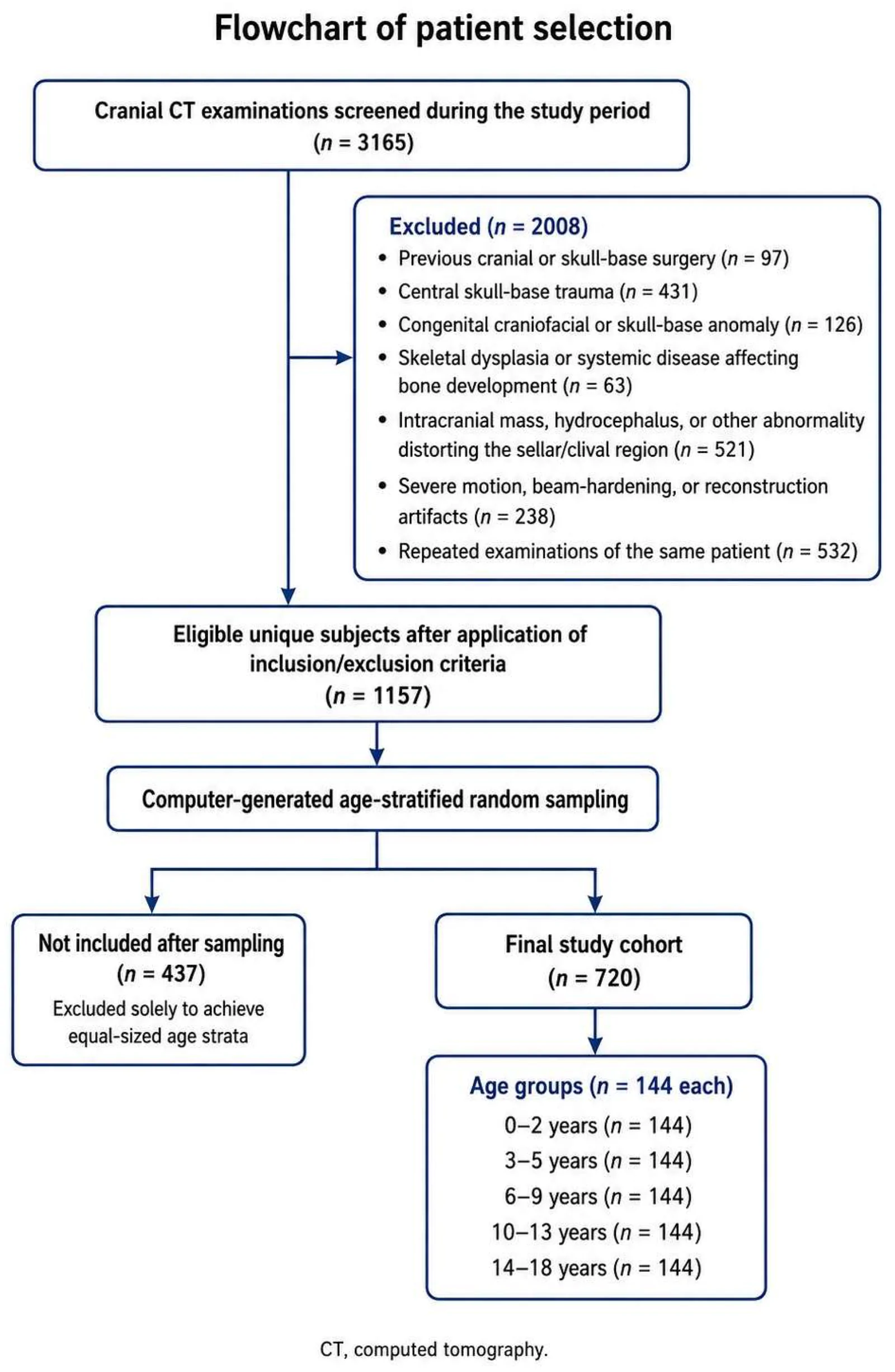
Flowchart of participant selection.

**Figure 2:**
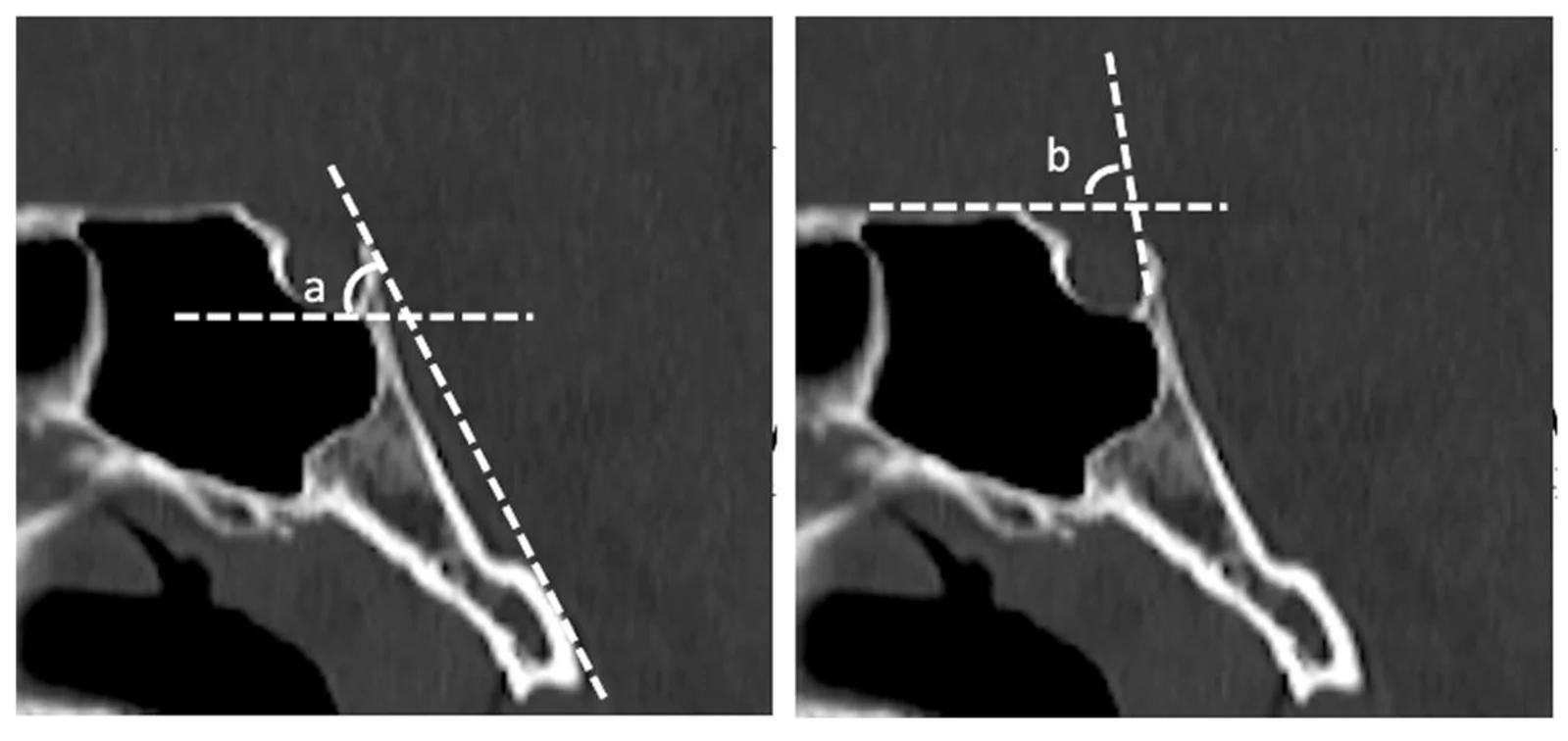
Angular relationships between the dorsum sellae and adjacent skull base structures: (**a**) sellar–clival inclination angle; (**b**) sphenoid–dorsal angle.

**Figure 3:**
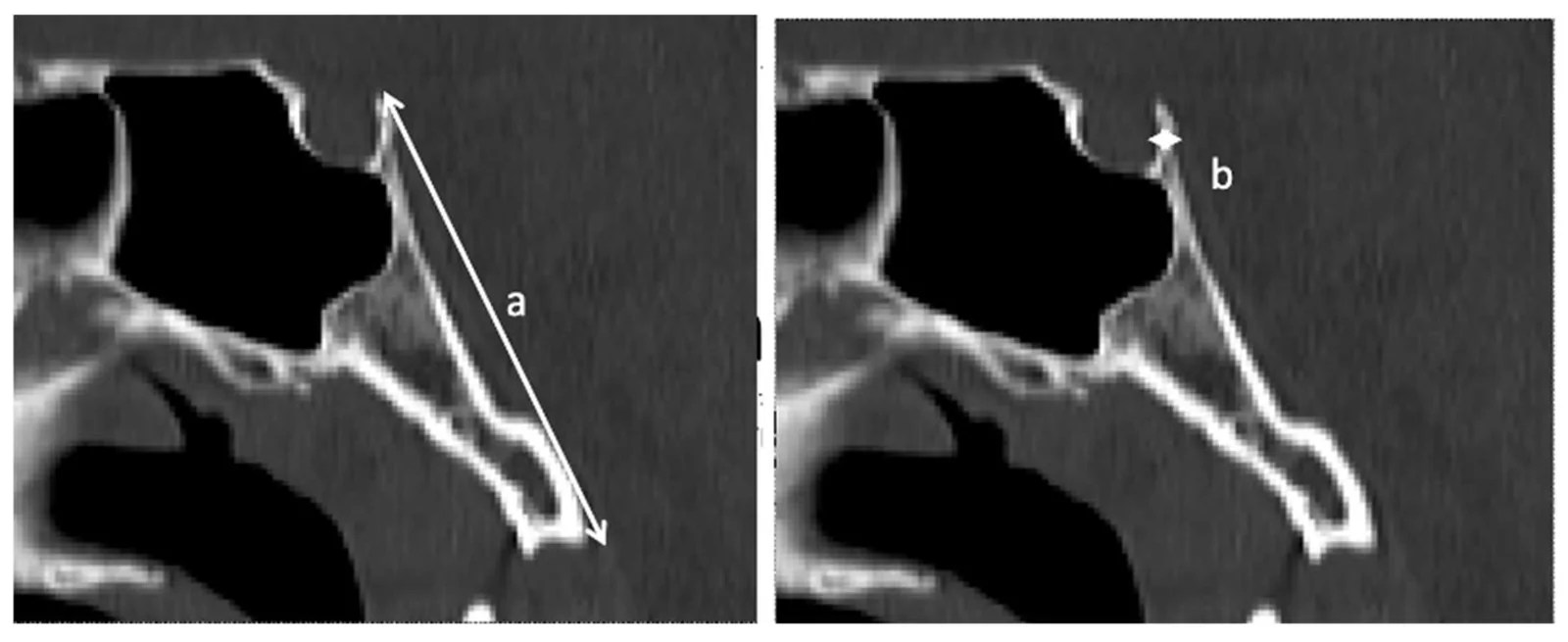
Linear measurements performed on midsagittal computed tomography images: (**a**) clival length as the linear distance from the dorsum sellae to the anterior margin of the foramen magnum along the clival surface; (**b**) middle thickness as the anteroposterior diameter of the dorsum sellae at its midpoint.

**Figure 4:**
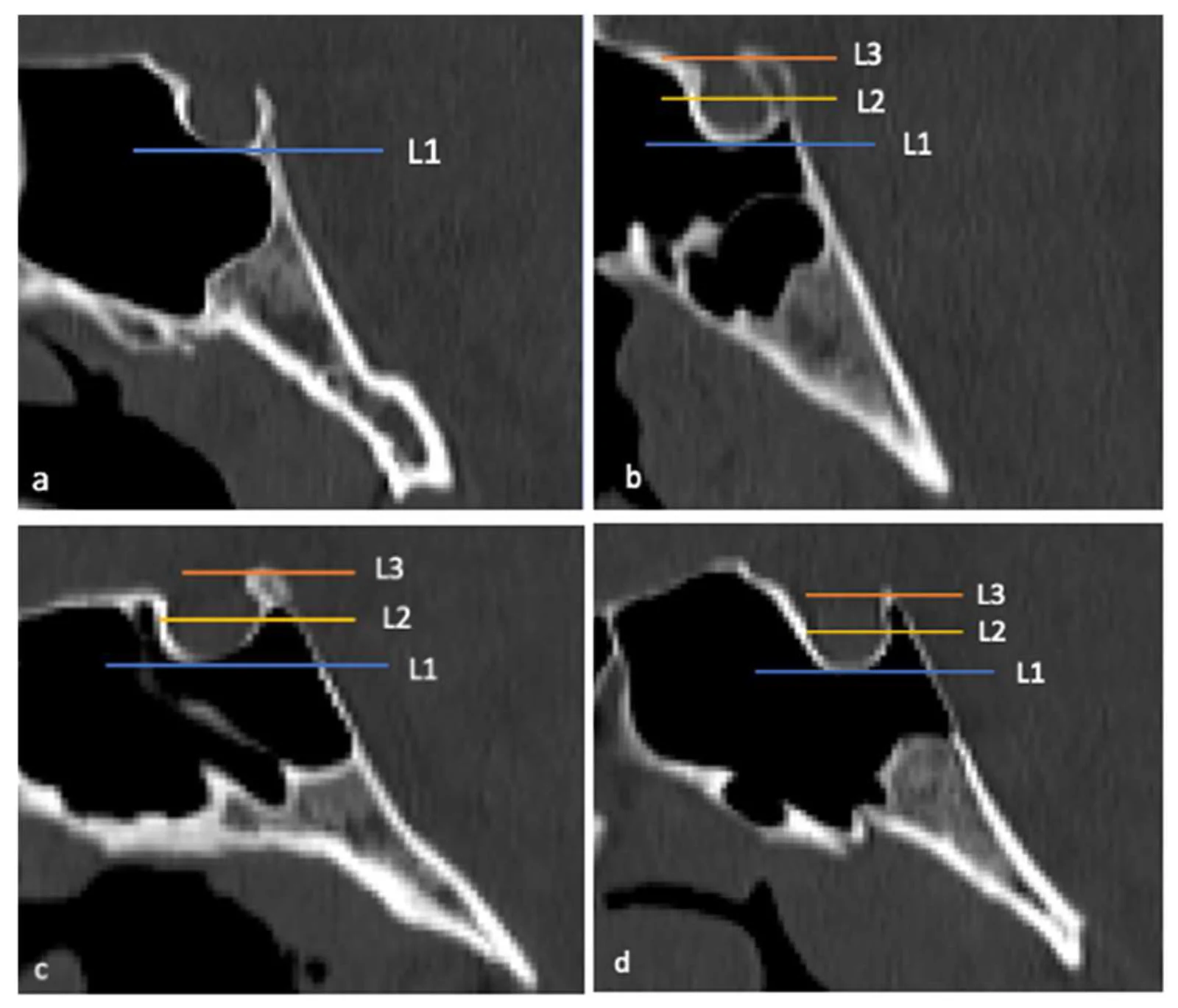
Dorsum sellae pneumatization patterns on midsagittal computed tomography images: (**a**) Type 0, no pneumatization; (**b**) Type 1, pneumatization <50% of the height of the dorsum sellae; (**c**) Type 2, pneumatization >50% without reaching the superior border; (**d**) Type 3, complete pneumatization extending to the superior border. L1, L2, and L3 indicate the lines passing through the inferior border, middle part, and superior tip of the dorsum sellae, respectively.

**Table 1:** Demographic characteristics.

Variable	Overall (*n* = 720)	Male (*n* = 396)	Female (*n* = 324)	*p*
Age (years), mean ± SD	7.87 ± 5.41	8.47 ± 5.50	7.13 ± 5.21	0.001
Age range	0–18	—	—	—
Sex, *n* (%)	720 (100%)	396 (55.0%)	324 (45.0%)	—
Age group 0–2	144	72 (50.0%)	72 (50.0%)	—
Age group 3–5	144	72 (50.0%)	72 (50.0%)	—
Age group 6–9	144	72 (50.0%)	72 (50.0%)	—
Age group 10–13	144	84 (58.3%)	60 (41.7%)	—
Age group 14–18	144	96 (66.7%)	48 (33.3%)	—

**Table 2:** Primary clinical indications for cranial CT.

Primary CT Indication	*n*	%
Head trauma	335	46.5
Headache	133	18.5
Suspected seizure	106	14.7
Altered level of consciousness	87	12.1
Vomiting	59	8.2
**Total**	**720**	**100.0**

CT, computed tomography. Each subject was assigned to a single primary indication based on the principal clinical reason documented in the CT request.

**Table 3:** Interobserver reliability.

Parameter	Interobserver ICC (95% CI)	Interpretation
Clivus length	0.999 (0.998–0.999)	Excellent
ML-T	0.986 (0.984–0.988)	Excellent
SDA	0.995 (0.994–0.996)	Excellent
SCIA	0.984 (0.981–0.986)	Excellent

ICC: Intraclass correlation coefficient; ML-T: Middle thickness of dorsum sellae; SDA: Sphenoid–dorsal angle; SCIA: Sellar–clival inclination angle.

**Table 4:** Morphometric measurements according to age group.

Parameter	0–2 Years (*n* = 144)	3–5 Years (*n* = 144)	6–9 Years (*n* = 144)	10–13 Years (*n* = 144)	14–18 Years (*n* = 144)	F	Holm-Adjusted *p*	ω^2^
Clivus length (mm)	32.11 ± 5.65	36.17 ± 2.27	42.74 ± 2.74	44.96 ± 2.91	46.96 ± 3.67	418.655	<0.001	0.699
ML-T (mm)	3.95 ± 0.40	3.89 ± 0.56	2.82 ± 0.43	2.39 ± 0.75	2.12 ± 0.61	323.135	<0.001	0.642
SDA (°)	103.49 ± 3.54	102.97 ± 4.31	93.11 ± 5.13	90.91 ± 4.23	89.95 ± 9.86	181.968	<0.001	0.501
SCIA (°)	58.18 ± 3.79	54.26 ± 4.69	58.03 ± 2.61	55.15 ± 3.65	57.56 ± 4.18	31.497	<0.001	0.145

Values are mean ± SD. F: ANOVA F-statistic; ω^2^: omega-squared (effect size). Reported *p* values for the four primary morphometric age-group comparisons are adjusted using the Holm–Bonferroni procedure.

**Table 5:** Unadjusted comparison of morphometric parameters by sex.

Parameter	Male (*n* = 396)	Female (*n* = 324)	Mean Difference	95% CI	Cohen’s d	*p*
Clivus length (mm)	41.28 ± 6.50	39.75 ± 6.79	1.52	[0.54, 2.50]	0.229	0.002
ML-T (mm)	3.03 ± 0.96	3.03 ± 0.93	0.00	[−0.14, 0.14]	−0.002	0.979
SDA (°)	95.57 ± 8.39	96.71 ± 8.27	−1.14	[−2.36, 0.08]	−0.137	0.069
SCIA (°)	56.56 ± 4.07	56.73 ± 4.31	−0.18	[−0.79, 0.44]	−0.042	0.576

Values are mean ± SD. Welch’s independent-samples *t*-test; these are crude comparisons without age adjustment.

**Table 6:** Correlation of morphometric parameters with age.

Parameter	Pearson r	95% CI	*p*
Clivus length (mm)	0.808	[0.781, 0.832]	<0.001
ML-T (mm)	−0.773	[−0.801, −0.742]	<0.001
SDA (°)	−0.663	[−0.702, −0.620]	<0.001
SCIA (°)	−0.005	[−0.078, 0.068]	0.884

**Table 7:** Secondary age- and sex-adjusted linear association analyses.

Outcome	Predictor	B	SE	Standardized β	95% CI	*p*	Adj R^2^
Clivus length	Age	1.044	0.042	0.847	[0.962, 1.127]	<0.001	0.653
	Sex	0.855	0.519	0.064	[−0.162, 1.872]	0.100	
	Age × Sex	−0.087	0.055	−0.077	[−0.195, 0.021]	0.116	
ML-T	Age	−0.135	0.006	−0.774	[−0.148, −0.123]	<0.001	0.605
	Sex	0.211	0.078	0.111	[0.057, 0.365]	0.007	
	Age × Sex	−0.004	0.008	−0.023	[−0.020, 0.013]	0.662	
SDA	Age	−1.040	0.067	−0.674	[−1.171, −0.909]	<0.001	0.437
	Sex	0.054	0.827	0.003	[−1.566, 1.674]	0.948	
	Age × Sex	0.024	0.088	0.017	[−0.148, 0.197]	0.781	
SCIA	Age	0.008	0.045	0.010	[−0.080, 0.095]	0.861	−0.004
	Sex	−0.038	0.552	−0.005	[−1.120, 1.043]	0.945	
	Age × Sex	−0.017	0.059	−0.024	[−0.133, 0.098]	0.767	

B: unstandardized regression coefficient; SE: standard error; β: standardized coefficient; Adj R^2^: adjusted R-squared. The models were used to estimate associations rather than to construct normative prediction equations; intercept-based individual predictions and prediction intervals were therefore not reported. The sex coefficient is adjusted for age and the age × sex interaction and should not be interpreted as equivalent to the crude mean difference in Table 5. A negative Adj R^2^ for SCIA indicates that age, sex, and their interaction jointly explained no meaningful variance in this parameter, consistent with the near-null linear correlation reported in Table 6. Sex was coded as 0 = female and 1 = male, and age was mean-centred before computing the age × sex interaction term.

**Table 8:** Distribution of dorsum sellae pneumatization types by age group.

Age Group	Type 0	Type 1	Type 2	Type 3	Total
0–2 years	144 (100.0%)	0 (0.0%)	0 (0.0%)	0 (0.0%)	144
3–5 years	144 (100.0%)	0 (0.0%)	0 (0.0%)	0 (0.0%)	144
6–9 years	144 (100.0%)	0 (0.0%)	0 (0.0%)	0 (0.0%)	144
10–13 years	74 (51.4%)	70 (48.6%)	0 (0.0%)	0 (0.0%)	144
14–18 years	95 (66.0%)	25 (17.4%)	16 (11.1%)	8 (5.6%)	144
Total	601 (83.5%)	95 (13.2%)	16 (2.2%)	8 (1.1%)	720

Pearson χ^2^ = 328.968; Fisher–Freeman–Halton exact test with Monte Carlo simulation (100,000 samples), *p* < 0.001; Cramer’s V = 0.390.

## Data Availability

The data presented in this study are available on request from the corresponding author due to institutional data protection restrictions.

## References

[1] Berger P.E., Harwood-Nash D.C., Fitz C.R. (1976). The dorsum sellae in infancy and childhood. Pediatr. Radiol..

[2] Gulsen S., Dinc A.H., Unal M., Cantürk N., Altinors N. (2010). Characterization of the anatomic location of the pituitary stalk and its relationship to the dorsum sellae, tuberculum sellae and chiasmatic cistern. J. Korean Neurosurg. Soc..

[3] Kumar T.S.M., Govindraju P. (2017). Relationship between the morphological variation of sella turcica with age and gender: A digital radiographic study. J. Indian Acad. Oral Med. Radiol..

[4] Atadağ A., Çetin C.Ş., Şencan Z., Artaş A., Aşantoğrol F., Sönmezışık S., Cihan Ö.F., Beger O. (2026). Development of the dorsum sellae in children: A CT study. Surg. Radiol. Anat..

[5] Alpergin B.C., Eroglu U., Gokalp E., Ozpiskin O.M., Gurses M.E., Akdoğdu B.S., Beger O. (2024). Pneumatization types of the dorsum sellae: A computed tomography study. Surg. Radiol. Anat..

[6] Ilków W., Waligóra M., Kunc M., Kucharzewski M. (2018). Pneumatization of the sphenoid sinus, dorsum sellae and posterior clinoid processes in computed tomography. Pol. J. Radiol..

[7] Alpergin B.C., Beger O., Zaimoglu M., Kılınç M.C., Özpişkin Ö.M., Erdin E., Çalışır E.S., Eroglu U. (2024). Posterior clinoid process in children: Morphometric analysis, pneumatization ratio, and surgical implications. Childs Nerv. Syst..

[8] Ji W., Kong G.G., Zheng M.H., Wang X.Y., Chen J.T., Zhu Q.A. (2015). Computed tomographic morphometric analysis of pediatric clival screw placement at the craniovertebral junction. Spine.

[9] Cheng Y., Chen Y., Zhou Z., Zhu J., Feng Y., Zhao G. (2015). Anatomical study of posterior clinoid process (PCP) and its clinical meanings. J. Craniofac. Surg..

[10] Krmpotić-Nemanić J., Vinter I., Kelović Z., Marušić A. (2005). Postnatal changes of the clivus. Ann. Anat..

[11] Hedayatian M., Azarbayejani S., Omrani A., Etemadi Borujeni S. (2024). Correlation of clivus length and angle with chronological age, gender, sagittal growth pattern of the jaws, and skeletal maturation using lateral cephalometry. J. Dent..

[12] Serindere G., Gunduz K., Avsever H. (2021). Morphological measurement and anatomical variations of the clivus using computed tomography. J. Neurol. Surg. B Skull Base.

[13] Chen J., Pool C., Slonimsky E., King T.S., Pradhan S., Wilson M.N. (2023). Anatomical parameters and growth of the pediatric skull base: Endonasal access implications. J. Neurol. Surg. B Skull Base.

[14] Burulday V., Akgül M.H., Muluk N.B., Ozveren M.F., Kaya A. (2017). Evaluation of posterior clinoid process pneumatization by multidetector computed tomography. Neurosurg. Rev..

[15] Jang Y.J., Kim S.C. (2000). Pneumatization of the sphenoid sinus in children evaluated by magnetic resonance imaging. Am. J. Rhinol..

[16] Szolar D., Preidler K., Ranner G., Braun H., Kern R., Wolf G., Stammberger H., Ebner F. (1994). Magnetic resonance assessment of age-related development of the sphenoid sinus. Br. J. Radiol..

[17] Ganesh V.L., Dharanipathy S., Pavana V., Kumar A., Sebastian L.J.D., Garg A. (2024). A computed tomography (CT)-based morphometric study of various skull base parameters and their anatomical relationships relevant to endoscopic endonasal skull base surgery. Surg. Neurol. Int..

[18] Banu M.A., Guerrero-Maldonado A., McCrea H.J., Garcia-Navarro V., Souweidane M.M., Anand V.K., Heier L., Schwartz T.H., Greenfield J.P. (2014). Impact of skull base development on endonasal endoscopic surgical corridors. J. Neurosurg. Pediatr..

[19] Valencia-Sanchez B.A., Kim J.D., Zhou S., Chen S., Levy M.L., Roxbury C., Patel V.A., Polster S.P. (2024). Special Considerations in Pediatric Endoscopic Skull Base Surgery. J. Clin. Med..

[20] Scuderi A.J., Harnsberger H.R., Boyer R.S. (1993). Pneumatization of the paranasal sinuses: Normal features of importance to the accurate interpretation of CT scans and MR images. AJR Am. J. Roentgenol..

[21] Yonetsu K., Watanabe M., Nakamura T. (2000). Age-related expansion and reduction in aeration of the sphenoid sinus: Volume assessment by helical CT scanning. AJNR Am. J. Neuroradiol..

[22] Vaezi A., Cardenas E., Pinheiro-Neto C., Paluzzi A., Branstetter B.F., Gardner P.A., Snyderman C.H., Fernandez-Miranda J.C. (2015). Classification of sphenoid sinus pneumatization: Relevance for endoscopic skull base surgery. Laryngoscope.

[23] Cellina M., Gibelli D., Floridi C., Toluian T., Valenti Pittino C., Martinenghi C., Oliva G. (2020). Sphenoid sinuses: Pneumatisation and anatomical variants—What the radiologist needs to know and report to avoid intraoperative complications. Surg. Radiol. Anat..

[24] Banu M.A., Rathman A., Patel K.S., Souweidane M.M., Anand V.K., Greenfield J.P., Schwartz T.H. (2014). Corridor-based endonasal endoscopic surgery for pediatric skull base pathology with detailed radioanatomic measurements. Neurosurgery.

